# Evaluation of High‐Density Lipoprotein Capacity to Acquire Free Cholesterol From Triglyceride‐Rich Lipoproteins During Reverse Remnant‐Cholesterol Transport in Familial Hypercholesterolemia

**DOI:** 10.1002/edm2.70269

**Published:** 2026-07-02

**Authors:** Shiva Ganjali, Judy B. de Haan, Wilfried Le Goff, Anatol Kontush, Amirhossein Sahebkar

**Affiliations:** ^1^ Department of Medical Biotechnology and Nanotechnology Mashhad University of Medical Sciences Mashhad Iran; ^2^ Cardiovascular Inflammation and Redox Biology Laboratory Baker Heart and Diabetes Institute Melbourne Victoria Australia; ^3^ Sorbonne Université, INSERM, Institute of Cardiometabolism and Nutrition (ICAN), UMR_S1166 Paris France; ^4^ Biotechnology Research Center, Pharmaceutical Technology Institute Mashhad University of Medical Sciences Mashhad Iran; ^5^ Centre for Research Impact & Outcome, Chitkara College of Pharmacy Chitkara University Rajpura Punjab India; ^6^ Applied Biomedical Research Center, Basic Sciences Research Institute Mashhad University of Medical Sciences Mashhad Iran

**Keywords:** familial hypercholesterolemia, HDL, reverse remnant‐cholesterol transport

## Abstract

**Introduction:**

High‐density lipoprotein (HDL) serves as a key protective factor against elevated low‐density lipoprotein cholesterol (LDL‐C) levels in patients with familial hypercholesterolemia (FH). This study aimed to assess whether HDL's ability to acquire free cholesterol (FC) from triglyceride‐rich lipoproteins (TGRLs) during lipolysis, as a part of reverse remnant‐cholesterol transport (RRT) pathway, is impaired in FH patients.

**Methods:**

This case–control study included 16 homozygous (HOFH) and 18 heterozygous (HEFH) patients with FH, along with 20 healthy controls. The free cholesterol transfer assay measured the ability of HDL (as apoB‐depleted serum) to uptake fluorescently labelled free cholesterol (TopF) from TGRLs during lipoprotein lipase (LPL)‐mediated lipolysis.

**Results:**

No significant difference was observed among the groups in the overall capacity of HDL to acquire FC from TGRLs during LPL‐mediated lipolysis. When normalizing for LDL‐C and non‐HDL‐C levels, significantly lower TopF transfer/LDL‐C and TopF transfer/non‐HDL‐C ratios were observed in HOFH patients in comparison with HEFH and healthy subjects. Lower TopF transfer/LDL‐C (OR: 0.605, 95% CI: 0.428–0.857; *p* < 0.01) and TopF transfer/non‐HDL‐C (OR: 0.539, 95% CI: 0.339–0.859; *p* < 0.01) ratios were independently associated with HOFH, while, after adjusting for age only the former association remained significant (OR: 0.646, 95% CI: 0.430–0.971).

**Conclusion:**

Although HDL's direct capacity to acquire free cholesterol from TGRLs did not differ significantly between the groups, the reduced TopF transfer/LDL‐C and TopF transfer non‐HDL‐C ratios in FH patients is consistent with impaired HDL function. These ratios may serve as a potential biomarker for identifying high‐risk FH phenotypes, warranting validation in larger, prospective studies.

## Graphical abstract

In familial hypercholesterolemia, HDL's absolute capacity to acquire free cholesterol from triglyceride‐rich lipoproteins during lipolysis is preserved, yet normalized HDL function indices relative to LDL‐C and non‐HDL‐C are markedly reduced in homozygous patients, suggesting these ratios as potential biomarkers for identifying high‐risk phenotypes where conventional lipid profiles fail to capture.

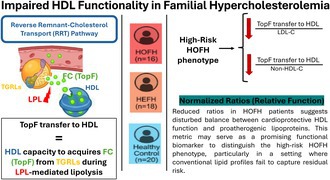

**Abbreviations**
apoA‐I: Apolipoprotein A‐I
apoB: Apolipoprotein B
ASCVD: Atherosclerotic cardiovascular disease
CE: Cholesteryl ester
CETP: Cholesteryl ester transfer protein
FC: Free cholesterol
FH: Familial hypercholesterolemia
HDL: High‐density lipoprotein
HEFH: Heterozygous FH
HOFH: homozygous FH
LCAT: Lecithin‐cholesterol acyltransferase
LDLR: Low‐density lipoprotein receptor
LPL: Lipoprotein lipase
mmol/L: Millimoles per litre
PCSK9: Proprotein subtilisin kexin 9
PL: Phospholipid
RCT: Reverse cholesterol transport
RRT: Reverse remnant‐cholesterol transport
SR‐BI: Scavenger receptor class B type I
TC: Total cholesterol
TG: Triglyceride
TGRL: Triglyceride‐rich lipoproteins
TopF: Fluorescently labelled free cholesterol (TopFluor cholesterol)
VLDL: Very low‐density lipoprotein

## 1 Introduction

Familial hypercholesterolemia (FH) is an autosomal dominant genetic disorder clinically characterized by markedly elevated plasma levels of low‐density lipoprotein cholesterol (LDL‐C) [1]. The most common form of FH is heterozygous FH (HEFH), which affects approximately 1 in 313 individuals and is caused by a single gene variant in genes involved in the *LDL receptor (LDLR), apolipoprotein B (apoB), or proprotein subtilisin kexin 9 (PCSK9)* pathways. The rare form of FH is homozygous FH (HOFH), with an estimated prevalence of 1 in 300,000 individuals, typically arising when a person inherits two pathogenic variants of the abovementioned genes [2]. Due to markedly elevated LDL‐C levels (> 13 mmol/L), individuals with HOFH are at a substantially increased risk of developing atherosclerotic cardiovascular disease (ASCVD) early in life. Consequently, early identification of FH cases and prompt initiation of lifelong lipid‐lowering therapy are critical to preventing premature ASCVD [2, 3]. Despite the technological advances in genetic diagnosis, FH is still underdiagnosed and undertreated [2, 4]. While the central role of elevated LDL‐C in the pathogenesis of atherosclerosis in FH is well established, increasing attention has focused on the contribution of high‐density lipoprotein (HDL) in modulating cardiovascular risk in these patients [5, 6, 7]. Both low HDL‐C levels and impaired HDL function have been associated with increased ASCVD risk in FH patients, independent of LDL‐C levels [8, 9, 10].

**FIGURE 1:**
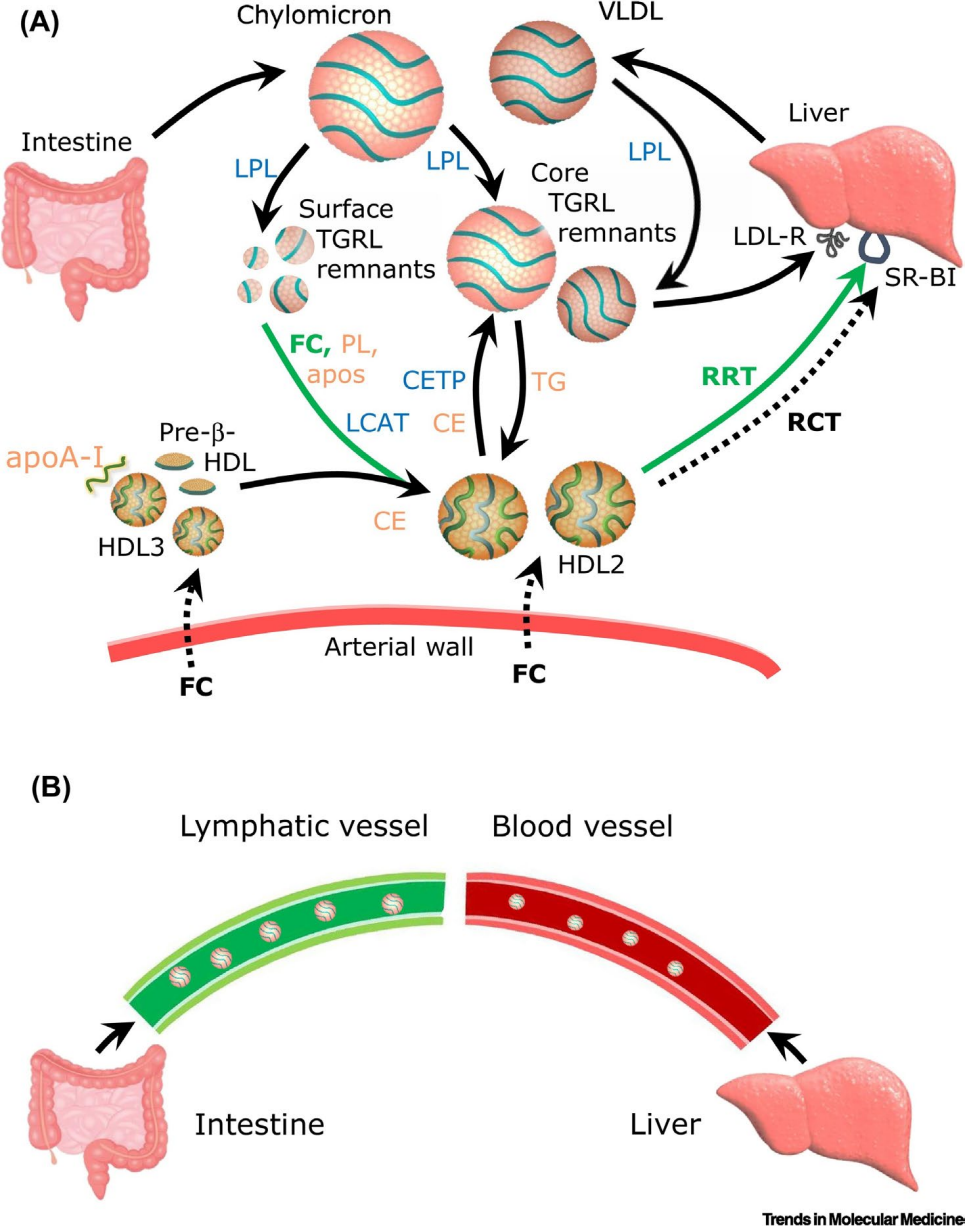
The Reverse Remnant‐Cholesterol Transport (RRT) Pathway. ApoA‐I: Apolipoprotein A‐I; CE: Cholesteryl ester; CETP: Cholesteryl ester transfer protein; FC: Free cholesterol; HDL: High‐density lipoprotein; LCAT: Lecithin‐cholesterol acyltransferase; LDL‐R: Low‐density lipoprotein receptor; LPL: Lipoprotein lipase; PL: Phospholipid; RCT: Reverse cholesterol transport; RRT: Reverse remnant‐cholesterol transport; SR‐BI: Scavenger receptor class B type I; TG: Triglyceride; TGRL: Triglyceride‐rich lipoproteins. VLDL: Very low‐density lipoprotein [12] (Licence Number: 6095320917813).

HDL is traditionally recognized for its protective role in reverse cholesterol transport (RCT), a process by which cholesterol is effluxed from peripheral tissues to the liver for excretion [6, 7, 11]. However, recent insights have extended this role to include a specific pathway called reverse remnant‐cholesterol transport (RRT). In this pathway, HDL actively participates in the clearance of cholesterol derived from triglyceride‐rich lipoproteins (TGRLs) such as chylomicron remnants and very‐low‐density lipoproteins (VLDL). As illustrated in Figure 1, TGRLs undergo progressive lipolysis in the circulation through the action of lipoprotein lipase (LPL), which hydrolyzes triglycerides and generates smaller, cholesterol‐enriched remnant particles. During this LPL‐mediated lipolysis, substantial surface components of TGRLs, comprising free cholesterol (FC), phospholipids (PL), and apolipoproteins, are shed and transferred to HDL particles. This process promotes the conversion of lipid‐poor pre‐β HDL into more mature HDL subclasses (HDL3 and HDL2). RRT represents a distinct but interconnected route of cholesterol flux, whereby HDL‐derived cholesterol, acquired from TGRL remnants rather than directly from peripheral cells, is ultimately delivered to the liver. As shown in the figure, this pathway operates alongside classical RCT from the arterial wall but specifically emphasizes the role of HDL in clearing remnant‐derived cholesterol from circulation. This process mitigates cholesterol accumulation in the arterial wall and may be particularly relevant in metabolic states characterized by elevated TGRLs or remnant cholesterol [12, 13]. Emerging data suggest that HDL function, especially its capacity to acquire FC during TGRL lipolysis, predicts CVD risk better than static HDL‐C levels alone [14]. Notably, studies have observed a U‐shaped relationship between HDL‐C concentrations and CVD risk, further underscoring the importance of HDL functionality over quantity [14, 15].

Despite this, the role of HDL‐mediated cholesterol transfer during RRT in FH has not been characterized. It remains unclear whether this specific HDL function is compromised in FH, particularly in relation to the severity of LDL‐C elevation. Therefore, this study aimed to evaluate the capacity of HDL to acquire FC upon lipolysis of TGRL by LPL in patients with FH.

## 2 Materials and Methods

### 2.1 Study Populations

In this case–controlled pilot study, 16 HOFH patients were included as a case group from all over Iran. Additionally, 18 HEFH participants were recruited from the family members (mother and/or father and/or siblings) of the HOFH patients. Each family contributed a maximum of two individuals, minimizing variability in family size and potential clustering effects. Selection criteria were defined previously [16, 17]. Also, 20 normolipidemic individuals were included and considered as the healthy control group.

### 2.2 Reagents

Phosphate‐buffered saline (PBS 10X including KCl) (10 g), KH_2_PO_4_ (10 g), Na_2_HPO_4_.2H_2_O (71.7 g), NaCl (400 g) (VWR, France); TopFluor cholesterol (TopF) (Avanti Polar Lipids, USA); lipoprotein lipase (LPL) from *Pseudomonas* sp. (Sigma, France), phosphotungstic acid (Merck, VWR), MgCl_2_ (VWR, France), deionized water.

### 2.3 ApoB Precipitation

A phosphotungstic acid/MgCl_2_ precipitation was used to remove apoB‐containing lipoproteins from fasted serum for the FC transfer assay. Phosphotungstic acid/MgCl_2_ (pH 6.2; 5 μL) was added to serum samples (50 μL) at a ratio of 1:10 by volume, incubated for 10 min at 20°C–22°C and centrifuged at 13,000 rpm at 4°C for 30 min (5415 R, Eppendorf, USA) [14].

### 2.4 TopF Cholesterol Transfer to HDL (TopF Transfer)

For the evaluation of HDL capacity to acquire FC through LPL‐mediated TGRL lipolysis, TGRLs (d < 1.019 g/mL) were obtained from a healthy normolipidemic subject via single‐step ultracentrifugation (40,000 rpm, 24 h, 15°C), dialysed against PBS (pH 7.4) using SERVAPOR dialysis tub (MWCO 12000–14,000 RC, 29 mm, Germany) at 4°C in the dark, and labelled with TopF. For the labelling, lipoprotein‐deficient plasma (LPDP) was added to the TGRL mixture at a 1:100 ratio (LPDP/TGRL) by volume and the mixture was filtered using a 0.8 μm filter. Chloroformic solution of TopF was added at a TopF/TGRL PL ratio of 1:13 by mass, and the mixture was incubated overnight at 37°C under gentle stirring. Unbound fluorescent lipid was separated from labelled TGRL by filtration through a PD‐10 Sephadex column (GE Healthcare, Sephadex G‐25 M, USA). Purified labelled TGRLs were assessed for TG concentration by photometry as well as, to verify the labelling, TopF fluorescence was recorded at the 500/525 nm (Ex/Em) using a microplate reader (Gemini, Molecular Devices, USA). To evaluate the labelling, 30 mg TG/dL of TopF‐labelled TGRLs, Tris buffer (0.4 M) (pH 8), a final dilution of HDL (as apoB‐depleted serum) 30‐fold v/v, and LPL (7600 U/L) were mixed on ice, and in order to start lipolysis, incubated at 37°C. After 2 h, the reaction mixture was placed on ice and apoB precipitant reagent (phosphotungstic acid/MgCl_2_) was added. The mixture was incubated for 10 min at RT, and centrifugated at 4°C for 10 min at a maximal speed (5415 R, Eppendorf). The supernatant containing the HDL was filtered (0.45 μm) and transferred to a black microplate (Corning, USA) in order to measure the HDL fluorescence. The fluorescence was expressed as the percentage of fluorescence (%TopF) in a standard sample containing TGRL (30 mg TG/dL) in Tris buffer and PBS alone. Finally, all values were normalized to that detected in HDL isolated by apoB precipitation from a reference serum sample obtained from a healthy normolipidemic control individual and expressed as a percentage of the latter (%TopF) [14].

Moreover, the TopF transferred to HDL was normalized to LDL‐C (TopF transfer/LDL‐C ratio), HDL‐C (TopF transfer/HDL‐C ratio), non‐HDL‐C (TopF transfer/non‐HDL‐C ratio) and very low‐density lipoprotein cholesterol (VLDL‐C) (TopF transfer/VLDL‐C ratio). Non‐HDL‐C was calculated as TC (mmol/L) minus HDL‐C. VLDL‐C was calculated as TG (mmol/L) divided by 2.2.

### 2.5 Statistics

For statistical analysis, SPSS software, version 11.5 (Chicago, IL, USA) was used. A *p*‐value less than 0.05 was defined as statistically significant. Continuous variables were assessed for normality using the one‐sample Kolmogorov–Smirnov test with exact significance values. All variables were normally distributed and are shown as mean ± standard error of mean (SEM). Differences in variables were analysed by one‐way analysis of variance (ANOVA) Tukey multiple comparison test across three groups (healthy, HEFH and HOFH) or a *t*‐test for independent samples between two groups (HEFH and HOFH, or FH (HOFH+HEFH) and the healthy group) where appropriate. Categorical variables are presented as percentages. Between‐group differences in categorical variables were evaluated by Chi squared analysis or a Fisher's exact test.

The correlation of TopF transfer to HDL with lipid profile and age was evaluated using Pearson's correlation coefficients. Multinomial and binary logistic regression was used to evaluate the association between TopF transfer to HDL and FH after adjustment for age.

## 3 Results

### 3.1 B*aseline Characteristics and Patient Status*

The HOFH group included significantly younger individuals than the two other groups. HEFH patients were also younger than healthy subjects (Table 1). In addition, HOFH patients demonstrated significantly elevated levels of total cholesterol (TC), triglyceride (TG), non‐HDL‐C, and LDL‐C in comparison with the two other groups. In contrast, HDL‐C concentrations showed no significant differences between the three groups (Table 1).

**TABLE 1:** Baseline characteristics of FH patients and controls.

Characteristics		Familial hypercholesterolemia (*N* = 34)		Healthy (*N* = 20)	*p*
		HEFH (*N* = 18)	HOFH (*N* = 16)		
Sex	Male	10 (55.6%)	5 (31.3%)	10 (50.0%)	0.361
	Female	8 (44.4%)	11 (68.8%)	10 (50.0%)	
Age (y)		33.6 ± 2.3^a^	14.0 ± 2.7	48.5 ± 1.9^a^, ^b^	< 0.001
TC (mmol/L)		6.4 ± 0.5^a^	16.1 ± 1.2	4.9 ± 0.2^a^	< 0.001
TG (mmol/L)		1.2 ± 0.1^a^	2.3 ± 0.4	1.4 ± 0.1^a^	< 0.01
HDL‐C (mmol/L)		1.6 ± 0.3	1.6 ± 0.2	1.3 ± 0.1	0.376
LDL‐C (mmol/L)		4.3 ± 0.4^a^	11.9 ± 1.1	2.9 ± 0.1^a^	< 0.001
VLDL‐C (mmol/L)		0.5 ± 0.0^a^	1.0 ± 0.2	0.6 ± 0.06^a^	< 0.01
Non‐HDL‐C (mmol/L)		4.5 ± 0.6^a^	14.6 ± 1.2	3.6 ± 0.2^a^	< 0.001

*Note:* Data are shown as mean ± SEM.

Abbreviations: HDL‐C, High‐density lipoprotein cholesterol; HEFH, Heterozygous familial hypercholesterolemia; HOFH, Homozygous familial hypercholesterolemia; LDL‐C, Low‐density lipoprotein cholesterol; mmol/L, Millimoles per litre; TC, Total cholesterol; TG, Triglyceride; VLDL‐C, Very low‐density lipoprotein cholesterol; y, Year.

^a^ Significant in comparison with HOFH group.

^b^ Significant in comparison with HEFH group.

Clinical characteristics of FH patients are summarized in Table 2. All patients in the HOFH group exhibited xanthomas and a majority of them (68.8%) experienced MI. FH score was expectedly higher in the HOFH relative to the HEFH group. Most of HEFH patients (66.7%) were not on drug therapy, whilst 33.3% were on statin therapy alone. In contrast, most of HOFH patients (75%) were on both statin and ezetimibe medication and 18.8% of them were on statin therapy alone (Table 2).

**TABLE 2:** Clinical characteristics of FH patients.

Variables		Familial hypercholesterolemia (*N* = 34)		*p*
		HEFH (*N* = 18)	HOFH (*N* = 16)	
FH score		15.0 ± 0.0	25.6 ± 0.5	< 0.001
Number of patients with xanthoma symptoms		0.0%	100.0%	< 0.001
Number of patients with MI history		0%	68.8%	< 0.001
Mutation (%)	Previously reported	84.6%	84.6%	1.000
	Novel	15.4%	15.4%	
Mutation type (%)	Missense	44.4%	50.0%	1.000
	Truncated	27.8%	25.0%	
	Single nucleotide variant	16.7%	12.5%	
	Single nucleotide polymorphism	11.1%	6.3%	
	Missense, truncated	0.0%	6.3%	
Position of LDLR mutation (%)	Exon	72.7%	84.6%	0.630
	Intron	27.3%	12.5%	
Drug consumption (%)	No drug	66.7%	6.2%	< 0.01
	Only statin	33.3%	18.8%	
	Statin + ezetimibe	0%	75.0%	

### 3.2 TopF Transfer to HDL

No significant differences in the capacity of apoB‐depleted serum to acquire FC from TGRL upon LPL‐induced lipolysis were found among the studied groups (Table 3). However, when normalizing for LDL‐C and non‐HDL‐C levels, significantly lower TopF transfer/LDL‐C and TopF transfer/non‐HDL‐C ratios were observed in HOFH patients in comparison with HEFH and healthy subjects. In addition, no significant differences in overall TopF transfer to HDL were found between the combined FH group (HEFH+HOFH) and healthy controls, while the TopF transfer/LDL‐C ratio showed significantly lower values in the combined FH group (HEFH+HOFH) in relation to healthy controls (19.1 ± 2.5 vs. 33.9 ± 2.9, *p* < 0.001), a difference predominantly attributable to the HOFH subgroup.

**TABLE 3:** TopF transfer to HDL from FH patients and controls.

Variables	Familial hypercholesterolemia (*N* = 34)		Healthy (*N* = 20)	*p*
	HEFH (*N* = 18)	HOFH (*N* = 16)		
TopF transfer to HDL (%)	102.8 ± 4.9	104.6 ± 5.5	95.3 ± 4.3	0.357
TopF transfer/HDL‐C	83.9 ± 9.6	77.8 ± 9.7	74.2 ± 4.4	0.690
TopF transfer/LDL‐C	28.7 ± 3.7^a^	10.1 ± 1.1	33.9 ± 2.9^a^	< 0.001
TopF transfer/VLDL‐C	218.1 ± 16.7	154.5 ± 23.4	183.4 ± 19.8	0.151
TopF transfer/non‐HDL‐C	33.6 ± 8.1^a^	8.2 ± 1.0	28.0 ± 2.2^a^	< 0.001

*Note:* Data are expressed as Mean ± SEM.

Abbreviations: HDL‐C, high‐density lipoprotein cholesterol; LDL‐C, Low‐density lipoprotein cholesterol; VLDL‐C, Very low‐density lipoprotein cholesterol.

^a^ Significant in comparison with HOFH group.

No correlation was found between TopF transfer to HDL and lipid profile, as well as age, in the studied groups (Table 4), even when FH (HOFH+HEFH) was considered as a single group. Multinomial logistic regression revealed that the reduced TopF transfer/LDL‐C (OR: 0.605, 95% CI: 0.428–0.857; *p* < 0.01) and TopF transfer/non‐HDL‐C (OR: 0.539, 95% CI: 0.339–0.859; *p* < 0.01) ratios were associated with HOFH. After adjusting for age as a confounder, only the association with TopF transfer/LDL‐C remained significant (OR: 0.646, 95% CI: 0.430–0.971; *p* < 0.05) (Table 5). Binary logistic regression showed no association of either HOFH (reference: HEFH) or FH (HEFH+HOFH) (reference: healthy) with measured variables, without or with adjustment for age (data not shown).

**TABLE 4:** The correlation of TopF transfer to HDL with lipid profile and age in studied groups.

	Studied groups	Correlations	Age	TC	TG	HDL‐C	LDL‐C	VLDL‐C	Non‐HDL‐C
TopF transfer to HDL	HOFH	Correlation	0.295	−0.332	−0.059	0.192	−0.131	−0.059	−0.359
		*p*	0.267	0.209	0.827	0.457	0.632	0.827	0.172
	HEFH	Correlation	0.157	−0.115	−0.011	−0.247	−0.316	−0.011	0.124
		*p*	0.535	0.682	0.973	0.438	0.251	0.973	0.701
	Healthy	Correlation	0.351	0.133	0.099	0.161	0.059	0.099	0.082
		*p*	0.129	0.577	0.679	0.498	0.805	0.679	0.732

Abbreviations: HDL‐C, High‐density lipoprotein cholesterol; HEFH, Heterozygous familial hypercholesterolemia; HOFH, Homozygous familial hypercholesterolemia; LDL‐C, Low‐density lipoprotein cholesterol; TC, Total cholesterol; TG, Triglyceride; VLDL‐C, Very low‐density lipoprotein cholesterol.

**TABLE 5:** Multinomial logistic regression for TopF transfer to HDL in relation to FH status (References: Healthy controls).

Variables	HOFH				HEFH			
	Unadjusted		Adjusted^#^		Unadjusted		Adjusted^#^	
	OR (95% CI)	*p*	OR (95% CI)	*p*	OR (95% CI)	*p*	OR (95% CI)	*p*
TopF transfer to HDL (%)	1.023 (0.989–1.058)	0.185	1.068 (0.993–1.148)	0.075	1.018 (0.986–1.052)	0.268	1.037 (0.983–1.094)	0.184
TopF transfer/HDL‐C	1.004 (0.982–1.027)	0.714	1.043 (0.985–1.105)	0.146	1.011 (0.987–1.035)	0.381	1.037 (0.988–1.088)	0.140
TopF transfer/LDL‐C	0.605 (0.428–0.857)	< 0.01	0.646 (0.430–0.971)	< 0.05	0.968 (0.913–1.026)	0.269	0.919 (0.824–1.025)	0.129
TopF transfer/VLDL‐C	0.995 (0.987–1.004)	0.289	0.996 (0.997–1.014)	0.636	1.005 (0.996–1.014)	0.272	1.005 (0.994–1.017)	0.372
TopF transfer/non‐HDL‐C	0.539 (0.339–0.859)	< 0.01	0.394 (0.074–2.093)	0.274	1.017 (0.976–1.060)	0.426	0.984 (0.927–1.045)	0.609

Abbreviations: CI, Confidence interval; HDL‐C, high‐density lipoprotein cholesterol; HEFH, Heterozygous familial hypercholesterolemia; HOFH, Homozygous familial hypercholesterolemia; LDL‐C, Low‐density lipoprotein cholesterol; OR, odds ratio; VLDL‐C, Very low‐density lipoprotein cholesterol.

^**#**^ Adjusted for age.

## 4 Discussion

This study demonstrates that the capacity of HDL to acquire FC during TGRLs lipolysis by LPL is impaired in FH when normalized to LDL‐C and non‐HDL‐C levels, with significantly reduced TopF transfer/LDL‐C and TopF transfer/non‐HDL‐C ratios observed in HOFH patients compared with HEFH patients and healthy controls. While no significant differences were found in overall TopF transfer to HDL across the groups, the reduced normalized ratios were strongly associated with the HOFH phenotype, and this association remained significant only for Topf transfer/LDL‐C ratio after adjustment for age. Previously, our team reported diminished HDL‐mediated cholesterol efflux in the RCT pathway in FH [5]. However, the ability of HDL to acquire FC via the RRT pathway has not been investigated in FH. Our findings suggest that while HDL‐C concentrations and absolute TopF transfer to HDL remain unchanged across groups, the reduced TopF transfer/LDL‐C ratio in HOFH patients indicates that HDL capacity to acquire FC is compromised in FH relative to severe LDL‐C burden characteristic of this phenotype. This distinction is important, as the reduced ratio may partly reflect the overwhelming LDL burden rather than exclusively representing intrinsic HDL dysfunction. It is well established that elevated LDL levels and proatherogenic environment can impair HDL functionality through several mechanisms, including alterations in the lipid composition of HDL particles and promotion of oxidative modification of HDL [18]. Nevertheless, the persistence of this association after adjustment for age suggests that the observed reduction is not merely a mathematical artefact of LDL burden alone, but may reflect a genuine impairment in the efficiency of HDL‐mediated cholesterol acquisition relative to the prevailing lipoprotein environment. The TopF transfer/LDL‐C ratio therefore captures the net functional capacity of HDL within the context of the overall atherogenic burden. This index may be more clinically informative than absolute transfer values alone, highlighting a core feature of FH‐related dyslipidemia that extends beyond conventional lipid metrics and may represent a relevant factor in FH clinical risk assessment and management [7, 19]. Previously, decreased transfer of FC to HDL as well as increased transfer of TG to HDL was observed in FH patients as compared to normolipidemic subjects under non‐lipolytic conditions, suggesting HDL instability and dysfunctionality in FH patients [20]. In the present study, elevated non‐HDL‐C and VLDL‐C levels in HOFH patients relative to HEFH patients and healthy controls may disturb the balance between proatherogenic and antiatherogenic lipoproteins [21], overwhelming the capacity of HDL to remove free cholesterol from TGRL. When we normalized TopF transfer to VLDL‐C level, the difference in this ratio did not reach significance across the three studied groups. However, normalizing the TopF transfer to non‐HDL‐C resulted in significantly lower TopF transfer/non‐HDL‐C ratio in HOFH patients compared with the two other control groups. Our data suggests that the balance of pro‐ and antiatherogenic factors may be compromised as a result of LDL‐C and non‐HDL‐C elevation in FH.

Importantly, our findings reveal that the relatively deficient HDL function persisted in the FH groups despite intensive lipid‐lowering therapy. Most of the HOFH patients were treated with statins and/or ezetimibe, yet they still exhibited a markedly reduced TopF transfer/LDL‐C ratio. This aligns with the previous evidence reporting that statin therapy may not fully restore HDL functionality, even though it effectively lowers LDL‐C [22]. In a subgroup analysis comparing patients with and without treatment, the former subgroup showed significantly lower normalized HDL function indices, including TopF transfer to HDL/LDL‐C (13.4 ± 1.7 vs. 31.1 ± 5.3), TopF transfer to HDL/non‐HDL‐C (11.0 ± 1.4 vs. 43.3 ± 12.9), and TopF transfer to HDL/VLDL‐C (160.2 ± 18.1 vs. 246.5 ± 21.3), compared with untreated individuals. Notwithstanding pharmacological treatment, LDL‐C levels remained significantly elevated in treated FH patients, most of whom had only recently been identified through cascade screening and were newly initiated on therapy. This likely reflects confounding by indication, whereby patients with the most severely elevated baseline LDL‐C were preferentially selected to receive treatment, as well as the limited capacity of statins and ezetimibe to fully compensate for the underlying receptor‐mediated defect in LDL clearance. Together, these observations suggest that treated patients represent a subgroup with more severe disease phenotype and higher residual cardiovascular risk despite therapy. In this context, the reduced ratios likely reflect impaired HDL function relative to an increased lipid burden rather than a treatment‐induced effect. This finding supports prior observations that remnant cholesterol and dysfunctional HDL both independently predict cardiovascular outcomes, even when LDL‐C is aggressively managed [23, 24, 25]. Consequently, functional impairment of HDL may represent a potential robust and clinically relevant marker of FH‐related dyslipidemia independent of HDL‐C concentration and treatment status. Moreover, in our study, TopF transfer to HDL did not show any correlations with lipid profile or age, reinforcing the notion that HDL functionality is a distinct and independent parameter that could be helpful for FH risk stratification beyond traditional lipid metrics.

A strength of this study is the use of a novel physiologically relevant assay to evaluate HDL function in the context of remnant lipoprotein metabolism, which contributes to cardiovascular risk in patients with FH [26, 27]. In contrast to standard cholesterol efflux assays that rely on macrophage cells and radiolabeled cholesterol, the use of fluorescently labelled TGRLs mimics the postprandial environment, enhancing both the practicality and translational relevance of our findings. Additionally, the inclusion of HEFH patients, who share lifestyle and genetic characteristics with the HOFH patients as a control group, represents another notable strength of this pilot investigation. However, several limitations must be acknowledged. First, the relatively small sample size, particularly in the HOFH group, limits statistical power and generalizability of the results, and the findings should therefore be interpreted as exploratory. Second, the cross‐sectional nature of this case–controlled study prevents causal inference regarding HDL function and FH severity. Third, healthy controls were not recruited from the same families as FH patients. Recruiting normolipidemic family members would have further controlled for shared lifestyle and genetic background; however, this was not feasible due to the limited availability of eligible normolipidemic relatives. Finally, the lack of clinical outcome data precluded the assessment of the association between HDL functional indices and cardiovascular events.

## 5 Conclusion

In conclusion, although HDL's capacity to acquire FC from TGRLs during lipolysis did not differ significantly between the groups, the reduced TopF transfer/LDL‐C and TopF transfer non‐HDL‐C ratios in FH patients suggest a disturbed balance between cardioprotective HDL function and proatherogenic lipoproteins. This metric may serve as a potential functional biomarker to distinguish the high‐risk FH phenotype, particularly in a setting where conventional lipid profiles fail to capture residual risk. This finding shifts, in part, focus from LDL‐C levels alone to the interplay between plasma lipoproteins, which can inform future research and risk assessment models for FH clinical management. Nevertheless, this hypothesis requires validation in larger, well‐designed prospective studies with adequately powered sample sizes and longitudinal follow‐up. Such studies should aim to evaluate the predictive value of HDL functional parameters in relation to clinically relevant endpoints, including cardiovascular events. This approach will help determine whether HDL dysfunction in the RRT pathway can serve as a clinically useful predictive biomarker for risk stratification and disease management in FH.

## Author Contributions

**Judy B. de Haan:** investigation, writing – review and editing. **Wilfried Le Goff:** investigation, writing – review and editing. **Anatol Kontush:** conceptualization, investigation, writing – review and editing. **Shiva Ganjali:** conceptualization, investigation, writing – original draft. **Amirhossein Sahebkar:** conceptualization, investigation, writing – review and editing.

## Funding

This work was supported by Mashhad University of Medical Sciences, 4000866.

## Ethics Statement

The study protocol was approved by the Ethics Committee of the Mashhad University of Medical Sciences (ID: IR.MUMS.REC.1400.200).

## Consent

Written informed consent was obtained from all participants or their parents.

## Conflicts of Interest

The authors declare no conflicts of interest.

## Data Availability

The data that support the findings of this study are available on request from the corresponding author. The data are not publicly available due to privacy or ethical restrictions.
